# Cancer stem cell markers in breast cancer: pathological, clinical and prognostic significance

**DOI:** 10.1186/bcr3061

**Published:** 2011-11-23

**Authors:** H Raza Ali, Sarah-Jane Dawson, Fiona M Blows, Elena Provenzano, Paul D Pharoah, Carlos Caldas

**Affiliations:** 1 Department of Oncology, University of Cambridge, Cambridge CB1 9RN, UK; 2 Cancer Research UK Cambridge Research Institute, Li Ka Shing Centre, Robinson Way, Cambridge CB2 ORE, UK; 3 Cambridge Breast Unit, Addenbrooke's Hospital, Cambridge University Hospital NHS Foundation Trust and NIHR Cambridge Biomedical Research Centre, Cambridge CB2 2QQ, UK; 4 Cambridge Experimental Cancer Medicine Centre (ECMC), Cancer Research UK Cambridge Research Institute, Li Ka-Shing Centre, Robinson Way, Cambridge CB2 0RE, UK; 5 Strangeways Research Laboratories, University of Cambridge, Cambridge CB1 9RN, UK; 6 Department of Histopathology, Addenbrooke's Hospital, Cambridge University Hospital NHS Foundation Trust, Cambridge CB2 2QQ, UK

## Abstract

**Introduction:**

The cancer stem cell (CSC) hypothesis states that tumours consist of a cellular hierarchy with CSCs at the apex driving tumour recurrence and metastasis. Hence, CSCs are potentially of profound clinical importance. We set out to establish the clinical relevance of breast CSC markers by profiling a large cohort of breast tumours in tissue microarrays (TMAs) using immunohistochemistry (IHC).

**Methods:**

We included 4, 125 patients enrolled in the SEARCH population-based study with tumours represented in TMAs and classified into molecular subtype according to a validated IHC-based five-marker scheme. IHC was used to detect CD44/CD24, ALDH1A1, aldehyde dehydrogenase family 1 member A3 (ALDH1A3) and integrin alpha-6 (ITGA6). A 'Total CSC' score representing expression of all four CSC markers was also investigated. Association with breast cancer specific survival (BCSS) at 10 years was assessed using a Cox proportional-hazards model. This study was complied with REMARK criteria.

**Results:**

In ER negative cases, multivariate analysis showed that ITGA6 was an independent prognostic factor with a time-dependent effect restricted to the first two years of follow-up (hazard ratio (HR) for 0 to 2 years follow-up, 2.4; 95% confidence interval (95% CI), 1.2 to 4.8; *P *= 0.009). The composite 'Total CSC' score carried independent prognostic significance in ER negative cases for the first four years of follow-up (HR for 0 to 4 years follow-up, 1.3; 95% CI, 1.1 to 1.6; *P *= 0.006).

**Conclusions:**

Breast CSC markers do not identify identical subpopulations in primary tumours. Both ITGA6 and a composite Total CSC score show independent prognostic significance in ER negative disease. The use of multiple markers to identify tumours enriched for CSCs has the greatest prognostic value. In the absence of more specific markers, we propose that the effective translation of the CSC hypothesis into patient benefit will necessitate the use of a panel of markers to robustly identify tumours enriched for CSCs.

## Introduction

The existence of tumour initiating cells also called cancer stem cells (CSCs) in breast cancer has been demonstrated by several studies [1-3]. It has been shown that xenotransplanted cell subpopulations enriched for CSCs can generate tumours in non-obese severe-combined immunodeficient (NOD/SCID) mice from a fraction of the number of unselected cells required to form tumours. In addition, tumours resulting from the implantation of small numbers of CSCs recapitulate the molecular heterogeneity of the original mixed population. The CSC hypothesis holds that since this subpopulation of cells is exclusively able to form tumours they underpin both disease recurrence and metastasis [4]. Therefore, CSCs are potentially of major clinical significance.

In order to demonstrate the functional characteristics which define a CSC, it is necessary to isolate candidate CSCs. This has been achieved by use of cell-surface markers and by tagging cells which exhibit characteristics associated with stemness. The combination of CD44 and CD24 first enabled Al-Hajj *et al*. to prospectively isolate a CSC subpopulation of from eight of nine patients with breast cancer [1]. After excluding non-epithelial cells (lineage^-^), CD44^+^CD24^-/low ^cells were enriched by flow cytometry and subsequently implanted into NOD/SCID mice. The CD44^+^CD24^-/low ^cells were able to form tumours in NOD/SCID mice from fewer cells than the mixed population with 10- to 50-fold enrichment for this ability. The resulting xenografts were found to exhibit the same phenotypic diversity as the original tumours [1].

A similar paradigm for experimentation was used to show that cell subpopulations with high aldehyde dehydrogenase (ALDH) activity were enriched for CSCs [3]. The ALDEFLUOR assay uses a biochemical reaction to tag cells with high ALDH activity with cytoplasmic fluorescence, permitting their enrichment by flow cytometry. Ginestier *et al*. found that ALDEFLUOR-positive normal mammary epithelial cells from reduction mammoplasties were enriched for sphere-forming ability and *in vivo *outgrowth potential, forming 10-fold more ducts in NOD/SCID mice. Similarly, ALDEFLUOR-positive cells from xenografts of human breast carcinomas were able to form tumours in NOD/SCID mice from as few as 500 cells, whereas ALDEFLUOR-negative cells inconsistently formed tumours and required 50, 000 cells to do so. Again, the tumours resulting from the implantation of ALDEFLUOR-positive cells contained both ALDEFLUOR-positive and negative cells in proportions similar to the original mixed population. The clinical relevance of this finding was investigated by using immunohistochemistry (IHC) to stain for aldehyde dehydrogenase family 1 member A1 (ALDH1A1) in 481 primary breast carcinomas. ALDH1A1 retained independent prognostic significance in a multivariate analysis [3]. The ALDEFLUOR assay is designed to detect expression of ALDH1A1 (STEMCELL Technologies SARL, Grenoble, France) and, consistent with this, Ginestier *et al*. found that ALDH1A1 expression was restricted to the ALDEFLUOR-positive subpopulation from normal mammary epithelial cells. However, the identity of the aldehyde dehydrogenase isoform(s) responsible for ALDEFLUOR-positivity in malignant breast epithelial cells has been questioned. Marcato *et al*. sought to establish whether ALDEFLUOR-positivity in primary breast tumours and breast cancer cell-lines related to a particular isoform(s) of ALDH or a global increase in ALDH activity [5]. Aldehyde dehydrogenase family 1 member A3 (ALDH1A3) not ALDH1A1, was found to correlate most strongly with ALDEFLUOR-positivity and, using immunofluorescence (IF) in primary tumours, was also found to correlate with metastasis and tumour grade. Moreover, the knockdown of ALDH1A3 in three breast cancer cell lines abrogated ALDEFLUOR activity [5].

An alternative approach to the CSC problem was used by Pece *et al.*, who, by exploiting the quiescent nature of normal mammary stem cells, isolated sufficient numbers to derive a gene expression signature [2]. The lipophilic dye PKH26 was used to isolate the most mitotically inactive fraction of self-renewing epithelial cells from reduction mammoplasties. The resulting gene signature was found to correlate with the grade of breast tumours. This correlation was established directly by comparison with published datasets and, indirectly, both by the prospective isolation of primary breast cancer cells using a subset of high-ranking markers from the gene signature and by IHC of breast tumours. By IHC and IF it was shown that grade 3 breast tumours contained a three- to four-fold greater proportion of cells expressing these high-ranking markers compared to grade 1 tumours. The authors argue that the grade of breast tumours is a function of their CSC content [2]. Prominent amongst the markers of the normal mammary stem cell-derived signature was CD49f or alpha-6 integrin (ITGA6). ITGA6 is a cell-surface protein which has been shown to identify adult mouse mammary stem cells [6] and a tumorigenic subpopulation in the MCF-7 breast cancer cell line [7] as well as regulating CSCs in glioblastoma [8].

Although CD44^+^CD24^-/low^, ALDH1A1, ALDH1A3 and ITGA6 appear to enrich for CSCs it is important to note that this is not always the case. For example, the CD44^+^CD24^-/low ^phenotype was not successful in identifying CSCs in one of the nine patient specimens originally reported [1]. Similarly, Hwang-Verslues *et al*. found that the expression of stem cell markers, including CD44^+^CD24^-/low ^and ALDH1A1, varied between breast cancer cell lines and between primary tumours, and that these markers did not universally enrich for CSCs [9]. Heterogeneity amongst the phenotype of CSCs and the existence of multiple clones of cells acting as CSCs are well-established concepts in the haematological malignancies [10]. It has been proposed that breast CSCs may exhibit heterogeneity between the subtypes of breast cancer in a manner analogous to the haematological malignancies [11].

Although several studies have profiled CSC markers in primary breast tumours [12-17], they have reached different conclusions and their precise clinical significance remains uncertain. We set out to establish the clinical relevance of the CSC hypothesis in breast cancer by profiling a large cohort of primary breast carcinomas using IHC and tissue microarrays (TMAs). We hypothesised that the significance of CSC markers may not be universal amongst breast cancers but may be subtype specific. In order to assess the relationship between subtype and CSC markers, we have divided tumours into molecular subtypes according to a validated panel of IHC markers and stratified all analyses by oestrogen receptor status (ER).

## Materials and methods

### Study population

The SEARCH breast study was used for this work. SEARCH is a large prospective population-based study of women diagnosed with breast cancer identified through the East Anglia Cancer Registry. It includes prevalent cases diagnosed before the age of 55 during 1991 to 1996 and still alive in 1996 and incident cases consisting of women under the age of 70 diagnosed after 1996; details of this study have been published previously [18]. A total of 4, 125 patients were included. Data on age at diagnosis, vital status including breast cancer-specific mortality, follow-up time, time between diagnosis and study entry, lymph node status, histological grade, tumour size, detection by mammographic screening, hormone therapy and chemotherapy were available. Details of the characteristics of the cohort are provided in Table 1. The SEARCH (Studies of Epidemiology and Risk Factors in Cancer Heredity) study is approved by the Cambridgeshire 4 Research Ethics Committee; all study participants provided written informed consent.

**Table 1:** Characteristics of the SEARCH study cohort

Variable		SEARCH	
Mean age (range)		52.6 (24 to 73)	
Mean follow-up in years (range)		8.5 (0.37 to 18.6)	
Number of breast cancer deaths (%)		563 (14)	
Five-year survival %		90	

	Categories	Number	Percent
Grade	1	768	19
	2	1, 622	39
	3	1, 020	25
	Missing	715	17
Node status	Negative	2, 309	56
	Positive	1, 424	35
	Missing	392	10
Tumour size	< 2 cm	2, 208	54
	2 to 4.9 cm	1, 522	37
	≥5 cm	145	4
	Missing	250	6
Endocrine therapy	No	770	19
	Yes	3, 315	80
	Missing	40	1
Chemotherapy	No	2, 692	65
	Yes	1, 392	34
	Missing	41	1
ER status	Negative	772	19
	Positive	2, 287	55
	Missing	1, 066	26
PR status	Negative	889	22
	Positive	2, 194	53
	Missing	1, 042	25
HER2 status	Negative	2, 560	62
	Positive	349	8
	Missing	1, 216	29
Molecular subtype	Luminal 1a	1, 631	40
	Luminal 1b	215	5
	Luminal 2	202	5
	HER2	120	3
	CBP	214	5
	5NP	130	3
	Missing	1, 613	39

### Immunohistochemistry and scoring

Paraffin embedded tissue blocks containing primary breast carcinoma were constructed as tissue microarrays (TMAs) as previously described [19]. Each tumour was represented by a 0.6 mm tissue core. Staining patterns in histologically normal breast tissue were assessed from one block. IHC was used to assay for the expression of cancer stem-cell related and other relevant proteins as detailed in Additional file 1. Briefly, 3 to 4 μm paraffin sections were dewaxed in xylene and rehydrated through graded alcohols. IHC was conducted using a BondMaX auto-immunostainer (Leica, Bucks, UK). Bound primary antibody was detected using a polymer-conjugated secondary antibody and staining was developed with 3-3'-diaminobenzidine (DAB). Double-immunostaining for detection of the CD44^+^CD24^-/low ^phenotype was done in sequence, with detection of bound mouse anti-CD24 antibody with a biotinylated secondary antibody developed with DAB and detection of bound rabbit anti-CD44 with a polymer-conjugated secondary antibody developed using alkaline phosphatase with fast-red as a chromogen. Stained TMAs were viewed following digitisation using the Ariol platform (Genetix Limited, Hampshire, UK). The extent of staining was assessed blinded to all patient and tumour characteristics. Only membranous CD44 expression was scored while cytoplasmic and apical staining of lumens was scored for CD24. For ALDH1A1 and ALDH1A3 only cytoplasmic staining was considered and expression by stromal cells was assessed separately. All CSC markers were scored by a pathologist (HRA) using an Allred scoring system accounting for both the intensity of staining (0 = none, 1 = weak, 2 = moderate, 3 = strong) and the proportion of stained cells (0 = 0%, 1 = < 1%, 2 = 1 to 10%, 3 = 11 to 33%, 4 = 34 to 66%, 5 = > 66%) producing a sum score of the two values (intensity + proportion = 0 to 8). The scoring system was chosen in consultation between HRA and a senior pathologist (EP). HRA has extensive experience in interpreting IHC in breast cancer TMAs, with Kappa agreement statistics of 0.81, 0.88 0.65 and 0.85 for the markers aurora kinase a (AURKA), Trans-acting T-cell-specific transcription factor GATA-3 (GATA3), mast/stem cell growth factor receptor kit (c-Kit) and DNA replication licensing factor MCM2 (MCM2) respectively. The cut-offs for scoring systems used for each antigen are detailed in Additional file 1. In order to address whether the combination of these markers offered superior prognostic value than their use separately, a "Total CSCs" variable was also created by adding the four dichotomised scores together to produce five categories. However, since only five cases were positive for all four markers, the four-marker-positive and three-marker-positive categories were merged leaving four categories (0 to 3).

### Definition of molecular subtype

Tumours were classified into six molecular subtypes using a validated IHC-based surrogate classifier according to the expression of ER, progesterone receptor (PR), human epidermal growth factor receptor 2 (HER2), cytokeratin 5/6 (CK5/6) and epidermal growth factor receptor (EGFR) [20]. Molecular subtypes were defined as: luminal 1a (ER+ or PR+, HER2-, CK5/6- and EGFR-), luminal 1b (ER+ or PR+, HER2-, CK5/6+ or EGFR+), luminal 2 (ER+ or PR+, HER2+), HER2 (ER- and PR-, HER2+), core basal phenotype (CBP) (ER- and PR-, HER2-, CK5/6+ or EGFR+) and 5-marker negative phenotype (5NP) (ER-, PR-, HER2-, CK5/6-, EGFR-).

### Statistical analyses

All analyses were stratified by ER status since ER expression defines fundamentally distinct diseases within breast cancer [20,21]. Correlations between ordinal variables were assessed using Spearman's rank correlation. Associations between categorical variables were assessed using Pearson's chi-square test or Fisher's exact test as appropriate. Associations with age were assessed using a Wilcoxon rank-sum test. A log-rank test was used to compare survival between groups in Kaplan-Meier survival plots. A Cox proportional-hazards model was used to investigate association with breast cancer-specific survival (BCSS) at 10 years follow-up, providing a hazard ratio (HR) and 95% confidence interval (95% CI) for each variable. Although the date of diagnosis was used to calculate time-to-event, since SEARCH is an ongoing study the date of study entry was used to determine time under observation in order to adjust for the bias of prevalent cases in a prospectively recruiting study (left-truncation) [22]. Likelihood ratios from univariate analyses were used to decide whether to model markers as continuous or dichotomised variables. Cut-points for dichotomisation were informed by comparing strata with non-expressing cases against BCSS in a Cox-proportional hazards model where there was no trend to hazard ratios, a pre-determined cut-point of > 2 was applied. Analyses exploring associations with clinical, molecular and survival data were also conducted using zero as a cut-point for dichotomisation of CSC markers in order to determine the extent to which patterns were dependent on different cut-points. Multivariate analyses were conducted for CSC markers significantly associated with BCSS on univariate analysis. Multivariate models were modified in a backward stepwise manner until the most parsimonious fit was attained. Covariates in the initial model included age (> 55 years), lymph node status, grade, tumour size (< 2 cm, 2 to 4.9 cm, ≥5 cm), endocrine therapy, adjuvant chemotherapy, PR and HER2 status. Grade, tumour size and 'Total CSCs' were modelled as continuous variables. Standard log-log plots were used to explore compliance with the Cox proportional-hazards assumption. For variables which violated the assumption, the Cox model was extended to include a coefficient which varied as a function of log-time, where if the HR decreases with time the log of the coefficient is < 1 and, conversely, > 1 if the HR increases with time. The *P*-value of the time-varying coefficient was also used to determine whether it was reasonable to model a variable as time-dependent in different subgroups. This work complied with reporting recommendations for tumour marker prognostic studies (REMARK) criteria [23]. All analyses were conducted using Intercooled Stata version 11.1 (StataCorp, College Station, TX, USA). All Stata command lines used to produce reported analyses can be made available on request. Heatmaps and dendograms for a single randomly selected imputed dataset using Allred scores were produced using Cluster [24] and Java TreeView as previously described [25].

### Missing data

The technical limitations of TMAs inevitably result in missing data. Tumour characteristics, such as size and morphology, tend to be correlated with the missingness of TMA data. Hence analyses, which exclude cases with missing data (complete case analysis (CCA)), can be biased [26]. In order to adjust for this source of bias we used multiple imputation (MI). MI is a method for handling missing data which has recently been validated for use in molecular pathology studies and been shown to produce more precise, less biased HRs compared to CCA [27]. MI generates a specified number of datasets wherein instances of missing data are resolved by randomly generated values which have been inferred under a model which takes account of the rest of the data. Subsequent analyses are performed on each imputed dataset and the results combined in a manner which accounts for the variability between imputed values. We used the *ice *command in Stata (StataCorp) to perform multiple imputation by chained equations [28,29] for 50 datasets across all IHC markers and relevant clinical variables including an outcome indicator (Nelson-Aalen estimator) to avoid inappropriate attenuation of associations [30]. Imputed data were then analysed using the *mi *commands. Results of survival analyses for both CCA and MI are presented for comparison.

## Results

### CSC markers have distinct expression patterns in normal and neoplastic breast tissue

**Figure 1:**
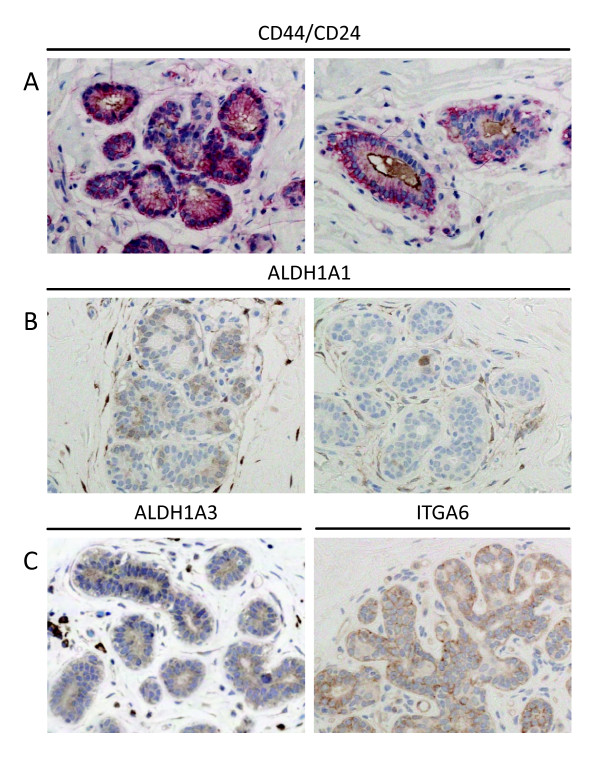
**Photomicrographs of CSC marker expression in normal breast tissue**. **A**. Double immunostaining for CD44 (red) and CD24 (brown) reveals membranous CD44 expression of myoepithelial cells and some luminal cells. CD24 stains luminal apical membranes and secretions. **B**. IHC for ALDH1A1 showed different patterns, including staining of single luminal cells (right panel), of whole lobules (left panel) and stromal cells. **C**. IHC for ALDH1A3 (left panel) shows weak cytoplasmic of most epithelial and mesenchymal cells. IHC for ITGA6 (right panel) shows membranous staining of both myoepithelial and luminal cells.

CSC markers showed distinct patterns of staining in normal breast tissue (Figure 1). Double-immunostaining for CD44^+^CD24^-/low ^revealed membranous CD44 expression primarily in myoepithelial cells, although there was also some expression by luminal cells. CD24 localised to the apical surface of luminal cells and also stained intra-luminal secretions. These patterns are consistent with those previously reported [14]. In keeping with the observations of Ginestier *et al*. [3], strong ALDH1A1 expression was seen in isolated luminal cells in terminal-ductal lobular units (TDLUs). However, in some TDLUs ALDH1A1 expression was observed more frequently, including occasional TDLUs where almost all cells were positive for ALDH1A1, again in keeping with staining patterns previously reported [14]. Myoepithelial cells were also observed to express ALDH1A1 both in ducts and, less often, in TDLUs. Nearly all stromal cells expressed ALDH1A1. ALDH1A3 was expressed very weakly in the cytoplasm of all mammary epithelial cells and stromal cells. For ITGA6, membranous staining of myoepithelial cells was predominant while staining of luminal cells was seen less frequently.

**Figure 2:**
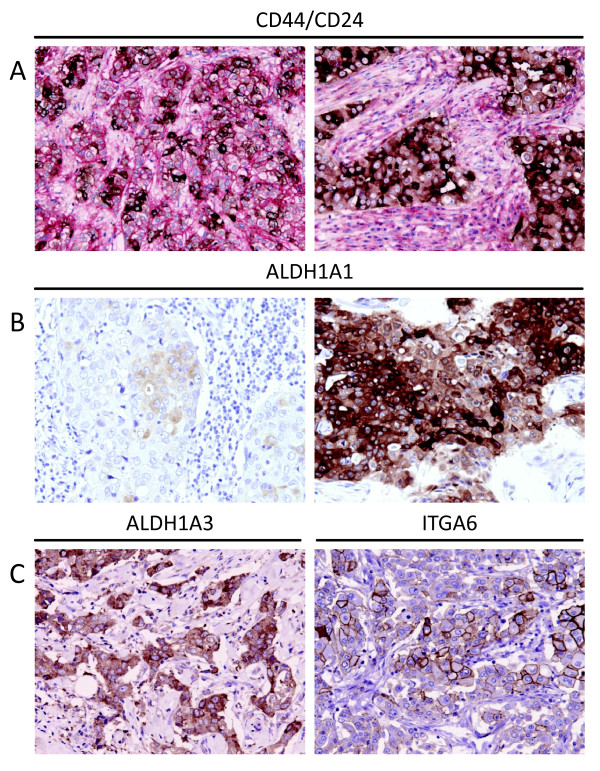
**Photomicrographs of CSC marker expression in invasive breast carcinoma**. **A**. Membranous CD44 (red) staining and cytoplasmic CD24 (brown) staining of carcinoma cells. Tumours contained different proportions of positive cells, including cases dominated by CD44^+^CD24^-/low ^cells (left panel) and others composed of CD24 expressing cells exclusively (right panel). **B**. Examples of low (left panel) and high (right panel) ALDH1A1 expression. **C**. Examples of high ALDH1A3 (left panel) and high ITGA6 (right panel) expression.

CSC markers were expressed at different levels in primary breast carcinomas (Figure 2 and Table 2). ALDH1A1 expression was least frequent amongst the CSC markers, with 59% of cases having an Allred score of 0 compared to ALDH1A3 expression where 43% of cases were scored as 0. There were more tumours with a maximum Allred score of 8 for the CD44^+^CD24^-/low ^phenotype than the other CSC markers (4% for CD44^+^CD24^-/low ^and ≤1% for the other CSC markers). There was a gradation of staining for all markers, ranging from single isolated cells to small clusters of cells to rare cases where all cells were strongly stained (Figure 2).

**Table 2:** Distribution of CSC marker scores

**Variable**		**Marker**									
		
		**CD44**^**+**^**CD24**^**-/low**^	**CD44**^**-**^**CD24**^**+**^	**CD44**^**+**^**CD24**^**+**^	**ALDH1A1**	**Stromal ALDH1A1**	**ALDH1A3**	**Stromal ALDH1A3**	**ITGA6**	**Score**	**Total CSCs**
		
Allred score	0	1, 808 (44)	814 (20)	1, 994 (48)	2, 452 (59)	1, 112 (27)	1, 763 (43)	2, 245 (54)	2, 004 (49)	0	1, 147 (28)
	2	27 (1)	18 (< 1)	34 (1)	169 (4)	303 (7)	43 (1)	12 (< 1)	20 (< 1)	1	401 (10)
	3	96 (2)	241 (6)	74 (2)	95 (2)	485 (12)	183 (4)	86 (2)	106 (3)	2	75 (2)
	4	103 (3)	318 (8)	174 (4)	69 (2)	442 (11)	162 (4)	102 (2)	77 (2)	3	18 (< 1)
	5	136 (3)	310 (8)	112 (3)	40 (1)	230 (6)	120 (3)	73 (2)	50 (1)	4	5 (< 1)
	6	103 (3)	358 (9)	52 (1)	27 (1)	156 (4)	194 (5)	64 (2)	39 (1)	Missing	2, 479 (60)
	7	104 (3)	375 (9)	74 (2)	20 (< 1)	130 (3)	119 (3)	27 (1)	49 (1)		
	8	160 (4)	103 (3)	23 (1)	18 (< 1)	32 (1)	27 (1)	2 (< 1)	44 (1)		
	Missing	1, 588 (39)	1, 588 (39)	1, 588 (39)	1, 235 (30)	1, 235 (30)	1, 514 (37)	1, 514 (37)	1, 736 (42)		
Cut-off		> 4	> 3	> 2	> 4	> 2	> 6	> 3	> 4		NA
Status*	Negative	2, 034 (80)	1, 073 (42)	2, 028 (80)	2, 785 (96)	1, 415 (49)	2, 465 (94)	2, 343 (90)	2, 207 (92)		
	Positive	503 (20)	1, 464 (58)	509 (20)	105 (4)	1, 475 (51)	146 (6)	268 (10)	182 (8)		

*Excluding missing cases. Percentages cited in parentheses

The correlations between CSC markers were stronger in ER- than ER+ disease (Additional file 2). In ER+ disease, ITGA6 was the only marker significantly correlated with all other CSC markers; it was most strongly correlated with ALDH1A3 with a Spearman's rho of 0.16, *P *< 0.0001. CD44^+^CD24^-/low ^was significantly correlated with ITGA6 only (Spearman's rho = 0.09, *P *= 0.0006). ALDH1A1 and ALDH1A3 were only weakly correlated in ER+ disease (Spearman's rho = 0.07, *P *= 0.0035). By contrast, in ER- disease all CSC markers were significantly positively correlated. The correlations between markers were also generally stronger in ER- cases. ITGA6 and CD44^+^CD24^-/low ^were the most strongly correlated markers (Spearman's rho = 0.29, *P *< 0.0001) while the weakest correlations were between ALDH1A1 and CD44^+^CD24^-/low ^(Spearmans's rho = 0.11, *P *= 0.0141) and between ALDH1A1 and ITGA6 (Spearmans's rho = 0.11, *P *= 0.0176).

### Association with clinical and molecular characteristics

CD44^+^CD24^-/low ^and ALDH1A1 expression were significantly associated with clinical features in analyses stratified by ER status (Table 3). In ER+ disease, CD44^+^CD24^-/low ^was associated with favourable clinical parameters. Of CD44^+^CD24^-/low ^positive tumours, 33% were grade 1 whereas only 23% of CD44^+^CD24^-/low ^negative tumours were grade 1 (*P *= 0.006). Similarly, 68% of CD44^+^CD24^-/low ^positive tumours were node negative, compared to 60% of CD44^+^CD24^-/low ^negative cases (*P *= 0.008). In ER+ disease CD44^+^CD24^-/low ^positive tumours were associated with ductal morphology with 81% of CD44^+^CD24^-/low ^positive cases being ductal compared to 73% of CD44^+^CD24^-/low ^negative cases (*P *= 0.012). ADLH1A1 positivity was significantly associated with high tumour grade in ER- disease only, with 43% of ALDH1A1 positive tumours being grade 3 compared to 20% of ALDH1A1 negative tumours (*P *= 0.012). In contrast to ER+ disease, the CD44^+^CD24^-/low ^phenotype was associated with higher tumour grade in ER- cases with 76% of positive tumours being grade 3 compared to 66% of negative cases (*P *= 0.020). However, as observed in ER+ disease, CD44^+^CD24^-/low ^positive tumours were more often node-negative in ER- cases also, with 64% of CD44^+^CD24^-/low ^positive tumours being node-negative compared to 51% of CD44^+^CD24^-/low ^negative cases (*P *= 0.012). In accordance with a putative CSC-marker, ALDH1A1 was significantly associated with positive lymph node status in ER- disease with 59% of ALDH1A1 positive cases being node positive compared to 43% of negative cases (*P *= 0.036). Notably, for analyses stratified by ER status, both ALDH1A3 and ITGA6 were not significantly associated with any clinical features.

**Table 3:** CSC marker associations with clinical characteristics

		ER POSITIVE								ER NEGATIVE							
**Variable**		**CD44**^**+**^**CD24**^**-/low**^		**ALDH1A1**		**ALDH1A3**		**ITGA6**		**CD44**^**+**^**CD24**^**-/low**^		**ALDH1A1**		**ALDH1A3**		**ITGA6**	
		**Negative**	**Positive**	**Negative**	**Positive**	**Negative**	**Positive**	**Negative**	**Positive**	**Negative**	**Positive**	**Negative**	**Positive**	**Negative**	**Positive**	**Negative**	**Positive**

Morphology	Ductal	1, 030 (73)	241 (81)	1, 357 (73)	38 (83)	1, 242 (73)	52 (75)	1, 116 (74)	41 (71)	369 (88)	125 (83)	491 (85)	45 (90)	434 (85)	60 (90)	373 (86)	83 (85)
	Lobular	246 (17)	34 (11)	315 (17)	3 (7)	277 (16)	11 (16)	241 (16)	11 (19)	20 (5)	6 (4)	32 (5)	0 (0)	25 (5)	3 (4)	23 (5)	4 (4)
	Other	140 (10)	23 (8)	188 (10)	5 (11)	176 (10)	6 (9)	142 (9)	6 (10)	32 (8)	19 (13)	58 (10)	5 (10)	52 (10)	4 (6)	37 (9)	11 (11)
	*P*-value	0.012		0.172		0.892		0.803		0.169		0.238*		0.625*		0.645*	
Grade	1	283 (23)	78 (33)	402 (25)	3 (11)	356 (25)	10 (19)	304 (24)	10 (26)	23 (6)	1 (1)	32 (6)	0 (0)	29 (7)	1 (2)	22 (6)	2 (3)
	2	684 (56)	112 (47)	873 (55)	13 (46)	787 (55)	28 (53)	700 (55)	20 (51)	103 (28)	28 (23)	145 (29)	7 (21)	119 (27)	11 (24)	109 (29)	15 (19)
	3	251 (21)	49 (21)	315 (20)	12 (43)	297 (21)	15 (28)	260 (21)	9 (23)	242 (66)	93 (76)	321 (64)	27 (79)	295 (67)	33 (73)	242 (65)	60 (78)
	*P*-value	0.006		0.012*		0.335		0.874		0.012*		0.164*		0.538*		0.086*	
Node status	Negative	783 (60)	184 (68)	1064 (61)	23 (53)	972 (62)	41 (63)	859 (62)	34 (63)	203 (51)	84 (64)	310 (57)	19 (41)	268 (56)	34 (54)	206 (52)	55 (62)
	Positive	533 (40)	86 (32)	669 (39)	20 (47)	602 (38)	24 (37)	528 (38)	20 (37)	195 (49)	48 (36)	231 (43)	27 (59)	207 (44)	29 (46)	194 (48)	34 (38)
	*P*-value	0.008		0.293		0.830		0.878		0.012		0.036		0.712		0.078	
Tumour size	< 2 cm	788 (58)	182 (64)	1050 (59)	22 (49)	964 (59)	37 (55)	842 (59)	30 (52)	176 (44)	61 (43)	236 (43)	23 (48)	208 (43)	23 (37)	167 (41)	38 (44)
	2 to 4.9 cm	545 (40)	88 (31)	695 (39)	22 (49)	628 (38)	28 (42)	551 (38)	27 (47)	208 (52)	74 (52)	289 (53)	23 (48)	259 (54)	36 (58)	225 (55)	45 (52)
	≥5 cm	33 (2)	13 (5)	50 (3)	1 (2)	44 (3)	2 (3)	44 (3)	1 (2)	14 (4)	6 (4)	24 (4)	2 (4)	16 (3)	3 (5)	20 (5)	3 (3)
	*P*-value	0.005		0.362*		0.747*		0.469*		0.916		0.777*		0.519*		0.821*	

*Fisher's exact test. Percentages are cited in parentheses.

**Figure 3:**
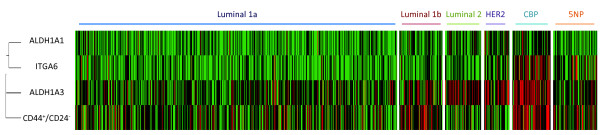
**Heatmap of CSC marker expression across breast cancer molecular subtypes**. Heatmap illustrating the unclustered distribution of cases from a single randomly selected imputed dataset across molecular subtypes defined by a five-marker IHC classifier. CSC markers arranged by average linkage clustering.

All CSC markers were significantly associated with negative ER and PR status (Additional file 3). ITGA6 positive tumours showed the strongest association with 63% of cases being ER-, compared to 22% of ITGA6 negative cases (*P *< 0.0001). Both ALDH1A1 and ALDH1A3 were associated with positive HER2 status where 26% of tumours positive for either marker were HER2 positive and 11% of negative cases were HER2 positive (*P *< 0.0001). By contrast, CD44^+^CD24^-/low ^positive cases were significantly associated with negative HER2 status (*P *= 0.025). These relationships were reflected in the pattern of association with molecular subtype (Table 4). The distribution of all CSC markers by molecular subtype is illustrated as a heatmap in Figure 3. Both CD44^+^CD24^-/low ^and ALDH1A3 were negatively associated with the luminal 1a subtype in both ER+ and ER- disease. The luminal subtypes in the ER- subgroup are ER-, PR+ tumours, of which there were 128. In ER- disease ALDH1A1 was also negatively associated with the luminal 1a subtype. Within ER- disease, we also found CD44^+^CD24^-/low ^to be associated with the CBP (basal) subtype consistent with previous reports [12]. There was a strong association between ALDH1A1 positivity and the HER2 subtype in ER- disease with 38% of ALDH1A1 positive tumours being of the HER2 subtype compared to 18% of ALDH1A1 negative cases (*P *= 0.001).

**Table 4:** CSC marker associations with molecular subtype and Ki67

		ER POSITIVE								ER NEGATIVE							
**Variable**		**CD44**^**+**^**CD24**^**-/low**^		**ALDH1A1**		**ALDH1A3**		**ITGA6**		**CD44**^**+**^**CD24**^**-/low**^		**ALDH1A1**		**ALDH1A3**		**ITGA6**	
																	
		**Negative**	**Positive**	**Negative**	**Positive**	**Negative**	**Positive**	**Negative**	**Positive**	**Negative**	**Positive**	**Negative**	**Positive**	**Negative**	**Positive**	**Negative**	**Positive**
Molecular subtype	Luminal 1a	1050 (83)	205 (75)	1, 331 (81)	28 (72)	1, 232 (81)	40 (59)	1, 045 (80)	41 (82)	60 (17)	9 (7)	75 (16)	2 (4)	71 (17)	2 (4)	62 (17)	8 (10)
	Other	219 (17)	69 (25)	307 (19)	11 (28)	283 (19)	28 (41)	257 (20)	9 (18)	292 (83)	120 (93)	408 (84)	43 (96)	359 (83)	54 (96)	294 (83)	69 (90)
	*P*-value	0.002		0.136		< 0.001		0.761		0.005		0.046*		0.009*		0.129	
	Luminal 1b	96 (8)	49 (18)	156 (10)	6 (15)	152 (10)	11 (16)	128 (10)	4 (8)	12 (3)	5 (4)	17 (4)	2 (4)	15 (3)	2 (4)	8 (2)	5 (6)
	Other	1173 (92)	225 (82)	1482 (90)	33 (85)	1363 (90)	57 (84)	1174 (90)	46 (92)	340 (97)	124 (96)	466 (96)	43 (96)	415 (97)	54 (96)	348 (98)	72 (94)
	*P*-value	< 0.001		0.221		0.103		0.812*		0.806		0.672*		1.000*		0.062*	
	Luminal 2	123 (10)	20 (7)	151 (9)	5 (13)	131 (9)	17 (25)	129 (10)	5 (10)	13 (4)	3 (2)	16 (3)	2 (4)	14 (3)	2 (4)	16 (4)	1 (1)
	Other	1, 146 (90)	254 (93)	1, 487 (91)	34 (87)	1, 384 (91)	51 (75)	1, 173 (90)	45 (90)	339 (96)	126 (98)	467 (97)	43 (96)	416 (97)	54 (96)	340 (96)	76 (99)
	*P*-value	0.215		0.444		< 0.001		0.983		0.576*		0.660*		0.705*		0.329*	
	HER2	NA		NA		NA		NA		81 (23)	14 (11)	86 (18)	17 (38)	83 (19)	14 (25)	76 (21)	12 (16)
	Other									271 (77)	115 (89)	397 (82)	28 (62)	347 (81)	42 (75)	280 (79)	65 (84)
	*P*-value									0.003		0.001		0.316		0.254	
	CBP	NA		NA		NA		NA		118 (34)	68 (53)	180 (37)	18 (40)	164 (38)	23 (41)	122 (34)	35 (45)
	Other									234 (66)	61 (47)	303 (63)	27 (60)	266 (62)	33 (59)	234 (66)	42 (55)
	*P*-value									< 0.001		0.717		0.671		0.064	
	5NP	NA		NA		NA		NA		68 (19)	30 (23)	109 (23)	4 (9)	83 (19)	13 (23)	72 (20)	16 (21)
	Other									284 (81)	99 (77)	374 (77)	41 (91)	347 (81)	43 (77)	284 (80)	61 (79)
	*P*-value									0.342		0.035*		0.489		0.913	
Ki67	Negative	1, 037 (77)	220 (77)	1, 370 (78)	27 (66)	1, 243 (78)	41 (61)	1, 088 (78)	37 (70)	196 (50)	52 (37)	262 (49)	13 (27)	232 (48)	23 (39)	200 (50)	30 (34)
	Positive	305 (23)	65 (23)	386 (22)	14 (34)	360 (22)	26 (39)	308 (22)	16 (30)	197 (50)	90 (63)	276 (51)	36 (73)	255 (52)	36 (61)	198 (50)	58 (66)
	*P*-value	0.977		0.064		0.002		0.163		0.007		0.003		0.208		0.006	

*Fisher's exact test. Percentages cited in parentheses

CSC markers were significantly associated with higher proliferation measured by Ki67 labelling (Table 4). ALDH1A3 positivity was associated with high Ki67 expression in ER+ disease, with 39% of ALDH1A3 positive cases having a Ki67 fraction of > 10% compared to 22% of ALDH1A3 negative cases (*P *= 0.002). In ER- disease, all CSC markers except ALDH1A3 were significantly associated with > 10% Ki67 staining. This relationship was strongest amongst ALDH1A1 positive tumours where 73% of positive cases were also Ki67 positive whereas just 51% of negative cases were Ki67 positive (*P *= 0.003). Associations with clinical and molecular characteristics for non-CSC markers (CD44^-^CD24^+^, CD44^+^CD24^+^, stromal ALDH1A1, stromal ALDH1A3) are detailed in Additional files 4 and 5.

### CSC markers predict poor outcome in ER- disease

There were 1, 127 cases with complete data for all relevant clinical variables and all IHC markers of a potential 4, 125 (27%). The median follow-up time was 8.54 years with a total of 740 deaths of which 563 were deaths from breast cancer. There were 507 deaths from breast cancer when follow-up was restricted to 10 years. Further details of the characteristics of the study cohort can be found in Table 1.

On univariate analysis, CSC markers showed distinct associations with survival and were more often associated with outcome in ER- disease (Additional files 6 and 7). The CD44^+^CD24^-/low ^phenotype was not significantly associated with survival. Although ALDH1A1 was associated with poor outcome in both ER+ (HR 2.5, 95% CI 1.1 to 5.6, *P *= 0.027) and ER- disease (HR 2.4, 95% CI 1.4 to 4.1, P = 0.002) when complete data were analysed, analysis of imputed data only reproduced the association within ER- disease (HR 1.9, 95% CI 1.1 to 3.2, *P *= 0.022) and not in ER+ cases (HR 1.6, 95% CI 0.73 to 3.6, *P *= 0.233). ALDH1A3 was significantly associated with survival within the ER- subgroup in both complete (HR 1.8, 95% CI 1.1 to 3.1, *P *= 0.026) and imputed (HR 1.7, 95% CI 1.1 to 2.9, *P *= 0.032) datasets. Similarly, ITGA6 was associated with poorer survival in ER- disease only. This association was time-dependent in both the complete and imputed data with the extended Cox-model showing that the hazard associated with ITGA6 positivity fell over time. The Total CSC score, representing a composite measure of all four CSC markers, also showed an association with poorer survival restricted to ER- disease and in the imputed dataset this effect was time-dependent with a reduction in hazard over time.

**Figure 4:**
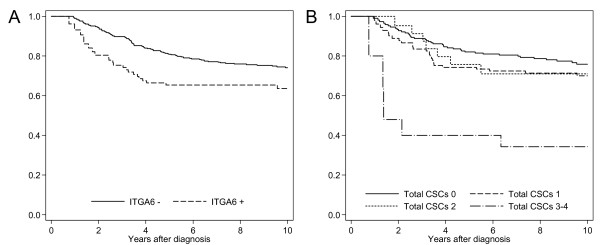
**Kaplan-Meier survival plots of ITGA6 and Total CSC expression in ER- cases for BCSS**. **A**. ITGA6 expression as a dichotomised variable (zero- to two-year follow-up, log-rank *P *= 0.0016; n (events): ITGA6- = 309 (14), ITGA6+ = 52 (8)). **B**. Total CSC composite score (zero- to four-year follow-up, log-rank *P *= 0.0173; n (events): CSC 0 = 164 (25), CSC 1 = 84 (22), CSC 2 = 19 (5), CSC 3 to 4 = 7 (4)).

On multivariate analysis both ITGA6 and the Total CSC composite score retained independent prognostic value in ER- disease (Table 5 and Figure 4). Multivariate analyses were restricted to CSC markers, which were associated with outcome on univariate analysis. ALDH1A1 showed independent prognostic value in ER- disease in CCA only. This effect was not reproduced when imputed data were analysed. ALDH1A3 was not significantly associated with outcome on multivariate analysis. As observed in univariate analyses, ITGA6 showed a time-dependent prognostic effect in both complete (HR 7.5, 95% CI 2.6 to 21.6, *P *< 0.001; T 0.18 95% CI 0.06 to 0.54, *P *= 0.002) and imputed (HR 2.8, 95% CI 1.2 to 6.3, *P *= 0.013; T 0.50, 95% CI 0.24 to 1.0, *P *= 0.055) datasets (CCA five-year BCSS adjusted for tumour size, grade and node status: ITGA6 negative = 87%; ITGA6 positive = 77%). In CCA the Total CSC variable was best modelled by not allowing for time-dependence. The Total CSCs composite score showed independent prognostic significance in complete data for ER- disease, conferring a 70% increased relative risk of event. In imputed data, the Total CSCs score also retained a significant association with survival for ER- disease and in a model allowing for time-dependence this effect diminished with time (HR 1.8, 95% CI 1.2 to 2.6, *P *= 0.002; T 0.71, 95% CI 0.51 to 0.99, *P *= 0.042). The five-year BCSS estimates for complete data adjusted for tumour size, grade and node status were Total CSCs = 0, 88%; Total CSCs = 1, 77%; Total CSCs = 2, 84%; Total CSCs = 3, 11%. Although for complete data the adjusted five-year survival is higher for a Total CSC score of 2 compared to 1, this was not reproduced in imputed data according to hazard ratios from a Cox proportional-hazards model where estimates of hazard increased successively with higher Total CSCs scores (data not shown). In order to investigate the relationship with survival time for ITGA6 and the Total CSCs score, follow-up time was divided into four periods (Table 6). Period-specific survival analyses showed that for ITGA6 adverse outcome associated with positivity was restricted to the first two years of follow-up, after which ITGA6 expression was not significantly associated with survival. Similarly, for the Total CSCs score unfavourable prognosis was restricted to the first four years after which there was no significant association with survival.

**Table 5:** Multivariate survival analyses of ER negative cases for ALDH1A1, ITGA6 and Total CSC score

Variable	Complete case analysis					Multiple imputation (M = 50)				
	n	HR (95% CI)	*P*	T (95% CI)	*P*	n	HR (95% CI)	*P*	T (95% CI)	*P*
Grade	481	3.6 (1.3 to 9.7)	0.011	0.32 (0.16 to 0.61)	0.001	1, 070	3.9 (1.8 to 8.3)	< 0.001	0.42 (0.25 to 0.68)	< 0.001
Node status		4.1 (2.6 to 6.3)	< 0.001	NA			2.8 (2.1 to 3.9)	< 0.001	NA	
Tumour size		*		NA			1.5 (1.1 to 1.9)	0.002	NA	
PR +		0.25 (0.13 to 0.50)	< 0.001	NA			0.16 (0.03 to 0.69)	0.015	2.1 (0.78 to 5.4)	0.146
HER2+		*		NA			1.4 (0.99 to 2.0)	0.060	NA	
**ALDH1A1 +**		**2.3 (1.3 to 4.0)**	**0.004**	**NA**			**1.4 (0.83 to 2.5)**	**0.191**	**NA**	
Grade	401	3.0 (0.99 to 9.0)	0.052	0.39 (0.18 to 0.82)	0.013	1, 070	4.2 (2.0 to 8.9)	< 0.001	0.40 (0.24 to 0.64)	< 0.001
Node status		4.2 (2.6 to 6.7)	< 0.001	NA			2.9 (2.1 to 4.0)	< 0.001	NA	
Tumour size		*		NA			1.5 (1.1 to 1.9)	0.003	NA	
PR +		0.23 (0.11 to 0.49)	< 0.001	NA			0.42 (0.26 to 0.69)	0.001	NA	
HER2+		*		NA			1.4 (1.0 to 2.0)	0.034	NA	
**ITGA6 +**		**7.5 (2.6 to 21.6)**	**< 0.001**	**0.18 (0.06 to 0.54)**	**0.002**		**2.8 (1.2 to 6.3)**	**0.013**	**0.50 (0.24 to 1.0)**	**0.055**
Grade	298	3.8 (1.1 to 13.0)	0.036	0.30 (0.13 to 0.68)	0.004	1, 070	4.0 (1.8 to 8.5)	< 0.001	0.41 (0.25 to 0.67)	< 0.001
Node status		4.2 (2.4 to 7.4)	< 0.001	NA			2.9 (2.1 to 4.0)	< 0.001	NA	
Tumour size		*		NA			1.5 (1.1 to 1.9)	0.003	NA	
PR +		0.15 (0.05 to 0.44)	< 0.001	NA			0.43 (0.26 to 0.70)	0.001	NA	
HER2 +		*		NA			1.4 (1.0 to 2.0)	0.037	NA	
Endocrine therapy		1.7 (0.98 to 2.9)	0.057	NA			*		NA	
**Total CSCs (0-3)**		**1.7 (1.3 to 2.4)**	**< 0.001**	**NA**			**1.8 (1.2 to 2.6)**	**0.002**	**0.71 (0.51 to 0.99)**	**0.042**

*Covariate dropped from final model. Grade, tumour size and Total CSCs modelled as continuous variables.

**Table 6:** Adjusted period-specific hazard ratios (95% CI) for ITGA6 and Total CSC

Marker	Follow-up (years)							
	**0 to 2**	***P*-value**	**2 to 4**	***P*-value**	**4 to 6**	***P*-value**	**6 to 10**	***P*-value**

ITGA6	2.4 (1.2 to 5.0)	0.019	1.4 (0.72 to 2.7)	0.329	0.60 (0.17 to 2.1)	0.429	0.68 (0.15 to 3.1)	0.612
Total CSCs	1.6 (1.1 to 2.3)	0.007	1.4 (1.0 to 1.9)	0.050	1.0 (0.60 to 1.7)	0.994	0.86 (0.43 to 1.7)	0.666

Adjusted for lymph node status, grade and tumour size. Analyses are of imputed data.

## Discussion

The CSC hypothesis holds that CSCs are solely responsible for tumour recurrence and metastasis [4]. The existence of CSCs in solid tumours was first demonstrated in breast cancer in 2003; since then other studies have also shown that a CSC population can be isolated from primary breast tumours [1-3]. The idea that CSCs are resistant to chemo- and radiotherapy has also been supported by some studies [31-33]. These findings are potentially of profound clinical importance and many attempts to understand their clinical relevance have been made. However, despite these efforts, the significance of CSCs remains uncertain and many questions persist. We have attempted to establish the clinical relevance of CSCs in breast cancer by using IHC to assay for putative CSC markers in a large cohort of primary breast tumours in TMAs. We find that CSC markers show distinct patterns of expression and association with clinical and molecular features. We also show that the prognostic significance of CSC markers is largely restricted to ER- disease and that the most robust predictor of outcome is a composite score representing expression of all four markers investigated. We show that this score is the most powerful predictor of outcome and an independent prognostic factor in ER- disease.

Our study has some potential limitations. First, since putative CSCs were originally identified using flow cytometry we have assumed that this assay can be reasonably translated into an IHC based equivalent. Although we would expect these modalities to identify a population with a high degree of overlap, it is probable that there will be some discordance. Second, we have used TMAs to detect a subpopulation of cells of reputed scarcity and as a result there is likely to be some sampling error. However, we have attempted to mitigate this effect by using a very large study cohort which has also enabled us to address important questions, especially those related to subtype, with statistical robustness. Finally, our analyses should be considered exploratory. Validation studies using identical methodology in independent cohorts are necessary before definitive conclusions can be drawn. Analyses of associations with clinical, molecular and outcome data where zero was used as a cut-point for dichotomisation are presented in Additional files 8, 9, 10 and 11. Most reported analyses are reproduced in these data, including the independent prognostic value of the 'Total CSC' score in the ER- subgroup. Although we find a small reduction in the hazard associated with CSC-positive cases treated with adjuvant chemotherapy compared to those who did not receive chemotherapy (data not shown), questions relating to the chemo-resistance of CSC-enriched tumours are best addressed in the context of randomised clinical trials.

The CD44^+^CD24^-/low ^phenotype was the first marker described to enrich for breast CSCs [1]. This prompted several attempts to characterise CD44^+^CD24^-/low ^cells in primary breast carcinomas. The prevalence of CD44^+^CD24^-/low ^cells has been shown to be associated with the basal-like subtype [12,14], to favour distant metastasis [34] and to be inversely associated with lymph node status [13]. An association with survival has been demonstrated by one study [15] and gene signatures derived from CD44+ primary breast cancer cells and CD44^+^CD24^- ^breast cancer cells (from xenografts or pleural effusions) have also been shown to correlate with outcome [35,36]. We also found that tumours enriched for the CD44^+^CD24^-/low ^phenotype were associated with the basal-like subtype and with negative lymph node status. In addition, we found that CD44^+^CD24^-/low ^tumours were associated with the luminal 1b subtype which, like basal-like tumours, is a subtype defined by basal cytokeratin expression. However, we did not find an association with survival.

Utilising the ALDEFLUOR assay, Ginestier *et al*. were able to use high aldehyde dehydrogenase activity as a basis for the enrichment of breast CSCs [3]. The group also found ALDH1A1 to be an independent prognostic factor when detected by IHC in primary breast carcinomas [3]. However, subsequent studies have not upheld the prognostic significance of ALDH1A1 [16,17]. Despite this and in keeping with the CSC hypothesis, ALDH1A1 has been found to predict response to chemotherapy [37]. We found that ALDH1A1 was an independent prognostic factor in ER- disease by CCA but that this finding was not reproduced when imputed data were analysed. Since missingness of data tends to be correlated across variables, estimates from CCA can be biased [26,27]. MI adjusts for this form of selection bias; hence, we consider estimates derived from MI more reliable than those from CCA. Our findings, coupled with those of other studies, imply that ALDH1A1 alone may not be a robust prognostic factor in breast cancer.

The assumption that ALDEFLUOR positivity of tumour cells correlates with ALDH1A1 expression by IHC has been questioned. Marcato *et al*. investigated which isoform of the aldehyde dehydrogenase family was most responsible for ALDEFLUOR positivity and found ALDH1A3 rather than ALDH1A1 to be the basis of ALDEFLUOR activity [5].

Although we found ALDH1A1 and ALDH1A3 to be positively correlated, the relationship was not strong (ER- cases, Spearman's rho = 0.19, *P *< 0.0001) and many cases showed discordant expression. We found ALDH1A3 to be significantly associated with survival in ER- disease on univariate analysis but this association was lost after adjustment for known prognostic factors in multivariate analysis.

ITGA6 expression has been linked to mammary stem cell biology in different ways. It has been used as a marker of murine mammary stem cells [5,6] and of tumorigenic cells of the MCF-7 breast cancer cell-line [7]. Pece *et al*. found ITGA6 to be highly expressed by normal human mammary stem cells and also showed that ITGA6 expression correlated with tumour grade [2]. Although we did not find a significant association between ITGA6 expression and higher tumour grade, there is a trend towards this in the ER- subgroup. ITGA6 expression has previously been shown to predict poor outcome in breast cancer [38]. We found ITGA6 to be an independent prognostic factor in ER- disease albeit restricted to the first two years of follow-up, after which ITGA6 expression was not associated with survival.

There is no highly specific marker for breast CSCs, rather the markers investigated in this study enrich tumour cell subpopulations for CSCs. We found a weak to moderate correlation between CSC markers, implying that different populations defined by these markers have some overlap but that most cells do not express these markers concurrently.

The idea of combining markers to increase the purity of subpopulations for CSCs was utilised by Ginestier *et al*. who showed that the combination of CD44^+^CD24^-/low ^and ALDEFLUOR activity enabled the isolation of cells able to form tumours in NOD/SCID mice from as few as 20 cells, compared to 500 cells when sorted by ALDEFLUOR activity alone [3]. Based on this finding, Neumeister *et al*. set out to establish the significance of combined CSC marker expression by investigating the expression of CD44 and ALDH1A1 in a cohort of 639 primary breast tumours [17]. They found no association with survival when they analysed the markers separately, but found the combination of the two markers to be an independent predictor of outcome [17]. Along these lines, we generated a score representing the sum of the dichotomised scores for all four markers. We found that this score was an independent prognostic factor in ER- disease.

## Conclusions

In summary, we have investigated the expression of putative CSC markers in a large cohort of primary breast carcinomas, treating ER+ and ER- tumours as distinct entities. We found that the patterns of association with clinical and molecular characteristics are different between CSC markers but that all markers were strongly associated with negative ER status. CSC markers did not carry significant prognostic value in ER+ tumours; therefore, additional markers enabling IHC quantification of CSCs in ER+ tumours (75 to 80% of all breast cancers) are required. In ER- disease, although only ITGA6 retained independent prognostic significance, a composite score representing expression of all four markers was the most powerful predictor of outcome. Based on our findings and in the absence of more specific CSC markers, we propose that it may be necessary to utilise a panel of CSC markers in order to effectively translate knowledge of CSCs into patient benefit.

## Abbreviations

ALDH1A1: retinal dehydrogenase 1; ALDH1A3: aldehyde dehydrogenase family 1 member A3; AURKA: aurora kinase a; BCSS: breast cancer-specific survival; CBP: core-basal phenotype; CCA: complete case analysis; CI: confidence interval; CK 5/6: cytokeratin 5/6; CSC: cancer stem cell; DAB: 3-3'-diaminobenzidine; EGFR: epidermal growth factor receptor; ER: estrogen receptor; HER2: human epidermal growth factor receptor 2; HR: hazard ratio; IF: immunofluorescence; IHC: immunohistochemistry; ITGA6: integrin alpha-6; MI: multiple imputation; NOD/SCID: non-obese severe combined immunodeficiency; PR: progesterone receptor; SEARCH: Study of Epidemiology and Risk Factors in Cancer Heredity; TDLU: terminal-ductal lobular unit; TMA: tissue microarray.

## Competing interests

The authors declare that they have no competing interests.

## Authors' contributions

HRA, EP, PDP and CC designed the study. HRA scored TMAs for CSC markers and conducted statistical analyses. SJD and EP scored TMAs for non-CSC markers. FMB constructed TMAs and compiled clinical data. PDP and CC are the project leaders for molecular pathology studies in SEARCH. All authors read and approved the final manuscript.

## Supplementary Material

Additional file 1 **Reagents and protocols for immunohistochemistry**. Click here for file

Additional file 2 **Contingency table for CSC markers by Spearman's rank correlation**. Click here for file

Additional file 3 **CSC marker associations with ER, PR and HER2**. Click here for file

Additional file 4 **Non-CSC marker associations with clinical characteristics**. Click here for file

Additional file 5 **Non-CSC marker associations with molecular characteristics**. Click here for file

Additional file 6 **Univariate survival analyses for all clinical and molecular markers (CCA)**. Click here for file

Additional file 7 **Univariate survival analyses for all clinical and molecular markers (MI)**. Click here for file

Additional file 8 **CSC marker associations with clinical characteristics using zero as a cut-point for dichotomisation**. Click here for file

Additional file 9 **CSC marker associations with molecular characteristics using zero as a cut-point for dichotomisation**. Click here for file

Additional file 10 **Univariate survival analyses of CSC markers using zero as a cut-point for dichotomisation**. Click here for file

Additional file 11 **Multivariate survival analyses for 'Total CSCs' using zero as a cut-point for dichotomisation of constituent CSC markers**. Click here for file
