# Supplementary material for: Cancer stem cell markers in breast cancer: pathological, clinical and prognostic significance
Source: Breast Cancer Res. 2011 Nov 23;13(6):R118. doi: 10.1186/bcr3061 (PMC3326560; doi:10.1186/bcr3061)
Supplement: Additional file 1 — Reagents and protocols for immunohistochemistry. [file bcr3061-S1.PDF]

Supplementary Tables

Supplementary Table 1: Reagents and protocols for immunohistochemistry

	Protein	Clone/Lot	Clonality	Source	Dilution	Antigen retrieval	Cut-off
**Molecular subtyping**	ER	6F11/2	Mouse monoclonal	Novocastra	1 in 70	Citrate buffer pH6, 30 minutes	>2
	PR	PgR636	Mouse monoclonal	Dako	1 in 50	Citrate buffer pH6, 30 minutes	>2
	HER2	c-erbB-2	Humanised monoclonal	Dako	1 in 250	Citrate buffer pH6, 40 minutes	≥ 2*
	CK5/6	D5/16 B4	Mouse monoclonal	Dako	1 in 50	Tris-EDTA buffer pH9, 30 minutes	>10%
	EGFR	31G7	Mouse monoclonal	Zymed	1 in 25	Proteinase K digestion, 10 minutes	≥ 2
**CSC markers**	CD44	B41535	Rabbit polyclonal	Atlas	1 in 200	Citrate buffer pH6, 30 minutes	>4
	CD24	SN3b	Mouse monoclonal	Thermo Fisher	1 in 200		
	ALDH1A1	R07299	Rabbit polyclonal	Atlas	1 in 50	Citrate buffer pH6, 20 minutes	>4
	ALDH1A3	RB16818	Rabbit polyclonal	Abgent	1 in 25	Citrate buffer pH6, 20 minutes	>6
	ITGA6	R06863	Rabbit polyclonal	Atlas	1 in 50	Tris-EDTA buffer pH9, 30 minutes	>4
**Other**	Ki67	MIB-1	Mouse monoclonal	Dako	1 in 200	Tris-EDTA buffer pH9, 30 minutes	>10%

*HercepTest™ :0 = No staining or weak staining in ≤ 10% of cells, 1 = Weak incomplete membranous staining in >10% of cells, 2 = Moderate circumferential membranous staining in > 10% of cells, 3 = Strong circumferential membranous staining in >10% of cells
