# Supplementary material for: Cancer stem cell markers in breast cancer: pathological, clinical and prognostic significance
Source: Breast Cancer Res. 2011 Nov 23;13(6):R118. doi: 10.1186/bcr3061 (PMC3326560; doi:10.1186/bcr3061)
Supplement: Additional file 2 — Contingency table for CSC markers by Spearman's rank correlation. [file bcr3061-S2.PDF]

Supplementary Table 2: Contingency table for CSC markers by Spearman's rank correlation

ER POSITIVE					ER NEGATIVE				
	$CD44^{+}CD24^{-/low}$	ALDH1A1	ALDH1A3	ITGA6		$CD44^{+}CD24^{-/low}$	ALDH1A1	ALDH1A3	ITGA6
$CD44^{+}CD24^{-/low}$	1				$CD44^{+}CD24^{-/low}$	1			
p-value	NA				p-value	NA			
ALDH1A1	0.04	1			ALDH1A1	0.11	1		
p-value	0.1247	NA			p-value	0.0141	NA		
ALDH1A3	0.03	0.07	1		ALDH1A3	0.13	0.19	1	
p-value	0.2286	0.0035	NA		p-value	0.0031	<0.0001	NA	
ITGA6	0.09	0.07	0.16	1	ITGA6	0.29	0.11	0.18	1
p-value	0.0006	0.0069	<0.0001	NA	p-value	<0.0001	0.0176	0.0002	NA
