# Supplementary material for: Cancer stem cell markers in breast cancer: pathological, clinical and prognostic significance
Source: Breast Cancer Res. 2011 Nov 23;13(6):R118. doi: 10.1186/bcr3061 (PMC3326560; doi:10.1186/bcr3061)
Supplement: Additional file 3 — CSC marker associations with ER, PR and HER2. [file bcr3061-S3.PDF]

Supplementary Table 3: CSC marker associations with ER, PR and HER2

Variable		$CD44^{+}CD24^{-/low}$		ALDH1A1		ALDH1A3		ITGA6	
		Negative	Positive	Negative	Positive	Negative	Positive	Negative	Positive
ER status	Negative	422 (23)	150 (33)	583 (24)	50 (52)	512 (23)	67 (49)	434 (22)	98 (63)
	Positive	1417 (77)	298 (67)	1862 (76)	46 (48)	1696 (77)	70 (51)	1499 (78)	58 (37)
p-value		<0.0001		<0.0001		<0.0001		<0.0001	
PR status	Negative	491 (26)	166 (37)	682 (27)	52 (51)	594 (26)	69 (51)	521 (26)	94 (57)
	Positive	1374 (74)	287 (63)	1811 (73)	49 (49)	1663 (74)	67 (49)	1453 (74)	72 (43)
p-value		<0.0001		<0.0001		<0.0001		<0.0001	
HER2 status	Negative	1588 (87)	408 (91)	2133 (89)	69 (74)	1943 (89)	100 (74)	1619 (87)	138 (87)
	Positive	228 (13)	39 (9)	270 (11)	24 (26)	239 (11)	36 (26)	236 (13)	21 (13)
p-value		0.025		<0.0001		<0.0001		0.860	
