# Supplementary material for: Cancer stem cell markers in breast cancer: pathological, clinical and prognostic significance
Source: Breast Cancer Res. 2011 Nov 23;13(6):R118. doi: 10.1186/bcr3061 (PMC3326560; doi:10.1186/bcr3061)
Supplement: Additional file 4 — Non-CSC marker associations with clinical characteristics. [file bcr3061-S4.PDF]

Supplementary Table 4: Non-CSC marker associations with clinical characteristics

		ER POSITIVE								ER NEGATIVE							
Variable		$CD44^-CD24^+$		$CD44^+CD24^+$		Stromal ALDH1A1		Stromal ALDH1A3		$CD44^-CD24^+$		$CD44^+CD24^+$		Stromal ALDH1A1		Stromal ALDH1A3	
		Negative	Positive	Negative	Positive	Negative	Positive	Negative	Positive	Negative	Positive	Negative	Positive	Negative	Positive	Negative	Positive
Morhology	Ductal	499 (70)	772 (77)	990 (72)	281 (83)	657 (72)	738 (74)	1183 (73)	111 (79)	194 (84)	300 (88)	372 (87)	122 (87)	282 (85)	254 (85)	403 (84)	91 (92)
	Lobular	150 (21)	130 (13)	252 (18)	28 (8)	170 (19)	148 (15)	272 (17)	16 (11)	15 (7)	11 (3)	22 (5)	4 (3)	20 (6)	12 (4)	27 (6)	1 (1)
	Other	63 (9)	100 (10)	135 (10)	28 (8)	84 (9)	109 (11)	168 (10)	14 (10)	21 (9)	30 (9)	36 (8)	15 (11)	31 (9)	32 (11)	49 (10)	7 (7)
	p-value	<0.001		<0.001		0.056		0.229		0.174		0.405*		0.463		0.073*	
Grade	1	185 (31)	176 (20)	296 (25)	65 (23)	221 (27)	184 (23)	345 (25)	21 (19)	12 (6)	12 (4)	23 (6)	1 (1)	20 (7)	12 (5)	29 (7)	1 (1)
	2	307 (52)	489 (57)	649 (55)	147 (52)	427 (53)	459 (57)	762 (55)	53 (49)	48 (25)	83 (28)	101 (27)	30 (26)	84 (29)	68 (28)	113 (27)	17 (22)
	3	100 (17)	200 (23)	231 (20)	69 (25)	158 (20)	169 (21)	278 (20)	34 (31)	134 (69)	201 (68)	249 (67)	86 (74)	183 (64)	165 (67)	269 (65)	59 (77)
	p-value	<0.001		0.184		0.087		0.017		0.452		0.038*		0.520		0.059*	
Node status	Negative	406 (62)	561 (60)	761 (60)	206 (65)	523 (62)	564 (61)	937 (62)	76 (57)	118 (59)	169 (51)	211 (54)	76 (56)	180 (58)	149 (54)	254 (57)	48 (51)
	Positive	247 (38)	372 (40)	506 (40)	113 (35)	322 (38)	367 (39)	569 (38)	57 (43)	83 (41)	160 (49)	183 (46)	60 (44)	131 (42)	127 (46)	189 (43)	47 (49)
	p-value	0.411		0.140		0.571		0.248		0.100		0.638		0.343		0.225	
Tumour size	<2cm	436 (64)	534 (55)	776 (59)	194 (60)	508 (57)	564 (59)	924 (59)	77 (55)	102 (48)	135 (41)	185 (46)	52 (39)	141 (45)	118 (41)	193 (43)	38 (40)
	2-4.9cm	231 (34)	402 (42)	512 (39)	121 (37)	352 (40)	365 (38)	596 (38)	60 (43)	104 (49)	178 (54)	206 (51)	76 (57)	155 (50)	157 (55)	240 (53)	55 (57)
	≥5cm	15 (2)	31 (3)	38 (3)	8 (2)	25 (3)	26 (3)	44 (3)	2 (1)	6 (3)	14 (4)	15 (4)	5 (4)	14 (5)	12 (4)	16 (4)	3 (3)
	p-value	0.002		0.846		0.772		0.434*		0.246		0.419		0.516		0.839*	

*Fisher’s exact test. Percentages cited in parentheses
