# Supplementary material for: Cancer stem cell markers in breast cancer: pathological, clinical and prognostic significance
Source: Breast Cancer Res. 2011 Nov 23;13(6):R118. doi: 10.1186/bcr3061 (PMC3326560; doi:10.1186/bcr3061)
Supplement: Additional file 5 — Non-CSC marker associations with molecular characteristics. [file bcr3061-S5.PDF]

Supplementary Table 5: Non-CSC marker associations with molecular characteristics

		ER POSITIVE								ER NEGATIVE							
Variable		$CD44^-CD24^+$		$CD44^+CD24^+$		Stromal ALDH1A1		Stromal ALDH1A3		$CD44^-CD24^+$		$CD44^+CD24^+$		Stromal ALDH1A1		Stromal ALDH1A3	
		Negative	Positive	Negative	Positive	Negative	Positive	Negative	Positive	Negative	Positive	Negative	Positive	Negative	Positive	Negative	Positive
Molecular subtype	Luminal 1a	524 (84)	731 (80)	1002 (82)	253 (80)	686 (85)	673 (77)	1182 (82)	90 (68)	24 (12)	45 (16)	62 (17)	7 (6)	47 (16)	30 (12)	68 (17)	5 (6)
	Other	100 (16)	188 (20)	224 (18)	64 (20)	122 (15)	196 (23)	268 (18)	43 (32)	174 (88)	238 (84)	301 (83)	111 (94)	240 (84)	211 (88)	334 (83)	79 (94)
	p-value	0.028		0.435		<0.001		<0.001		0.244		0.003		0.203		0.011	
	Luminal 1b	62 (10)	83 (9)	115 (9)	30 (9)	62 (8)	100 (12)	148 (10)	15 (11)	10 (5)	7 (2)	15 (4)	2 (2)	11 (4)	8 (3)	12 (3)	5 (6)
	Other	562 (90)	836 (91)	1111 (91)	287 (91)	746 (92)	769 (88)	1302 (90)	118 (89)	188 (95)	276 (98)	348 (96)	116 (98)	276 (96)	233 (97)	390 (97)	79 (94)
	p-value	0.550		0.964		0.008		0.697		0.132		0.264*		0.752		0.178	
	Luminal 2	38 (6)	105 (11)	109 (9)	34 (11)	60 (7)	96 (11)	120 (8)	28 (21)	8 (4)	8 (3)	13 (4)	3 (3)	8 (3)	10 (4)	14 (3)	2 (2)
	Other	586 (94)	814 (89)	1117 (91)	283 (89)	748 (93)	773 (89)	1330 (92)	105 (79)	190 (96)	275 (97)	350 (96)	115 (97)	279 (97)	231 (96)	388 (97)	82 (98)
	p-value	<0.001		0.315		0.011		<0.001		0.465		0.771*		0.390		1.0*	
	HER2	NA		NA		NA		NA		23 (12)	72 (25)	78 (21)	17 (14)	47 (16)	56 (23)	76 (19)	21 (25)
	Other									175 (88)	211 (75)	285 (79)	101 (86)	240 (84)	185 (77)	326 (81)	63 (75)
	p-value									<0.001		0.093		0.048		0.204	
	CBP	NA		NA		NA		NA		88 (44)	98 (35)	115 (32)	71 (60)	112 (39)	86 (36)	149 (37)	38 (45)
	Other									110 (56)	185 (65)	248 (68)	47 (40)	175 (61)	155 (64)	253 (63)	46 (55)
	p-value									0.030		<0.001		0.430		0.161	
	5NP	NA		NA		NA		NA		45 (23)	53 (19)	80 (22)	18 (15)	62 (22)	51 (21)	83 (21)	13 (15)
	Other									153 (77)	230 (81)	283 (78)	100 (85)	225 (78)	190 (79)	319 (79)	71 (85)
	p-value									0.284		0.112		0.902		0.279	

*Fisher's exact test. Percentages cited in parentheses
