# Supplementary material for: Cancer stem cell markers in breast cancer: pathological, clinical and prognostic significance
Source: Breast Cancer Res. 2011 Nov 23;13(6):R118. doi: 10.1186/bcr3061 (PMC3326560; doi:10.1186/bcr3061)
Supplement: Additional file 6 — Univariate survival analyses for all clinical and molecular markers (CCA). [file bcr3061-S6.PDF]

Supplementary Table 6: Univariate survival analyses for all clinical and molecular markers (CCA)

Complete Case Analysis										
Variable	ER Positive					ER Negative				
	n	HR (95% CI)	P	T (95% CI)	P	n	HR (95% CI)	P	T (95% CI)	P
Grade	1926	2.2 (1.8 - 2.7)	<0.001	NA		646	6.0 (2.5 - 14)	<0.001	0.29 (0.16 - 0.52)	<0.001
Tumour size	2157	2.2 (1.8 - 2.8)	<0.001	NA		703	1.8 (1.4 - 2.4)	<0.001	NA	
Node Status	2063	3.6 (2.7 - 4.9)	<0.001	NA		700	3.6 (2.5 - 5.1)	<0.001	NA	
Endocrine therapy	2243	0.84 (0.55 - 1.3)	0.436	NA		757	0.94 (0.69 - 1.3)	0.714	NA	
Chemotherapy	2243	6.0 (2.5 - 14.1)	<0.001	0.53 (0.30 - 0.92)	0.023	756	1.9 (1.3 – 2.6)	<0.001	NA	
PR	2125	0.49 (0.35 - 0.69)	<0.001	NA		704	0.04 (0.01 - 0.23)	<0.001	4.8 (1.6 - 14.8)	0.006
HER2	2000	2.2 (1.5 - 3.3)	<0.001	NA		638	1.6 (1.1 - 2.3)	0.011	NA	
$CD44^{+}CD24^{-/low}$	1681	0.62 (0.39 - 1.0)	0.057	NA		561	1.1 (0.76 - 1.7)	0.539	NA	
$CD44^{-}CD24^{+}$	1681	1.6 (1.1 - 2.1)	0.007	NA		561	0.41 (0.19 - 0.92)	0.030	2.4 (1.2 - 4.7)	0.011
$CD44^{+}CD24^{+}$	1681	0.53 (0.33 - 0.85)	0.009	NA		561	0.60 (0.37 - 0.97)	0.036	NA	
ALDH1A1	1873	2.5 (1.1 - 5.6)	0.027	NA		621	2.4 (1.4 - 4.1)	0.002	NA	
Stromal ALDH1A1	1873	0.77 (0.58 - 1.0)	0.071	NA		621	0.99 (0.70 - 1.4)	0.940	NA	
ALDH1A3	1731	1.5 (0.74 - 3.1)	0.260	NA		566	1.8 (1.1 - 3.1)	0.026	NA	
Stromal ALDH1A3	1731	0.74 (0.38 - 1.45)	0.383	NA		566	1.6 (1.0 - 2.5)	0.055	NA	
ITGA6	1523	0.85 (0.32 - 2.3)	0.755	NA		519	4.7 (1.8 - 12.1)	0.002	0.32 (0.14 - 0.74)	0.008
Total CSCs	1123	0.98 (0.68 - 1.4)	0.930	NA		375	1.3 (1.0 - 1.7)	0.027	NA	
