# Supplementary material for: Cancer stem cell markers in breast cancer: pathological, clinical and prognostic significance
Source: Breast Cancer Res. 2011 Nov 23;13(6):R118. doi: 10.1186/bcr3061 (PMC3326560; doi:10.1186/bcr3061)
Supplement: Additional file 7 — Univariate survival analyses for all clinical and molecular markers (MI). [file bcr3061-S7.PDF]

Supplementary Table 7: Univariate survival analyses for all clinical and molecular markers (MI)

Variable	Multiple Imputation (M=50)									
	ER Positive					ER Negative				
	n	HR (95% CI)	P	T (95% CI)	P	n	HR (95% CI)	P	T (95% CI)	P
Grade	2903	5.0 (2.7 - 9.3)	<0.001	0.57 (0.38 - 0.84)	0.005	1070	5.6 (2.7 - 11.7)	<0.001	0.38 (0.23 - 0.61)	<0.001
Tumour size		2.3 (1.9 - 2.8)	<0.001	NA			1.8 (1.4 - 2.3)	<0.001	NA	
Node Status		3.2 (2.5 - 4.1)	<0.001	NA			3.2 (2.4 - 4.4)	<0.001	NA	
Endocrine therapy		0.87 (0.59 - 1.3)	0.469	NA			1.0 (0.75 - 1.4)	0.927	NA	
Chemotherapy		8.1 (3.8 - 17.3)	<0.001	0.46 (0.28 - 0.75)	0.002		2.2 (1.6 - 2.9)	<0.001	NA	
PR		0.53 (0.39 - 0.73)	<0.001	NA			0.10 (0.02 - 0.4)	0.002	2.9 (1.1 - 7.5)	0.030
HER2		2.1 (1.4 - 3.0)	<0.001	NA			1.6 (1.2 - 2.3)	0.004	NA	
$CD44^{+}CD24^{-/low}$		0.73 (0.48 - 1.1)	0.152	NA			1.1 (0.76 - 1.6)	0.605	NA	
$CD44^{-}CD24^{+}$		1.4 (1.0 - 1.8)	0.026	NA			1.1 (0.82 - 1.5)	0.456	NA	
$CD44^{+}CD24^{+}$		0.67 (0.43 - 1.0)	0.062	NA			0.79 (0.51 - 1.2)	0.296	NA	
ALDH1A1		1.6 (0.73 - 3.6)	0.233	NA			1.9 (1.1 - 3.2)	0.022	NA	
Stromal ALDH1A1		0.89 (0.68 - 1.2)	0.386	NA			1.1 (0.82 - 1.5)	0.477	NA	
ALDH1A3		1.3 (0.69 - 2.6)	0.394	NA			1.7 (1.1 - 2.9)	0.032	NA	
Stromal ALDH1A3		1.0 (0.56 - 1.8)	0.996	NA			1.7 (1.1 - 2.6)	0.015	NA	
ITGA6		0.90 (0.39 - 2.1)	0.817	NA			3.0 (1.3 - 6.8)	0.008	0.45 (0.22 - 0.92)	0.029
Total CSCs		0.94 (0.70 - 1.3)	0.684	NA			2.1 (1.4 - 3.1)	<0.001	0.63 (0.46 - 0.88)	0.007
