# Supplementary material for: Cancer stem cell markers in breast cancer: pathological, clinical and prognostic significance
Source: Breast Cancer Res. 2011 Nov 23;13(6):R118. doi: 10.1186/bcr3061 (PMC3326560; doi:10.1186/bcr3061)
Supplement: Additional file 8 — CSC marker associations with clinical characteristics using zero as a cut-point for dichotomisation. [file bcr3061-S8.PDF]

Supplementary Table 8: CSC marker associations with clinical characteristics using zero as a cut-point for dichotomisation

		ER POSITIVE								ER NEGATIVE							
Variable		CD44$^+$CD24$^{-/low}$		ALDH1A1		ALDH1A3		ITGA6		CD44$^+$CD24$^{-/low}$		ALDH1A1		ALDH1A3		ITGA6	
		Negative	Positive	Negative	Positive	Negative	Positive	Negative	Positive	Negative	Positive	Negative	Positive	Negative	Positive	Negative	Positive
Morphology	Ductal	923 (72)	348 (80)	1206 (73)	189 (73)	916 (72)	378 (76)	1032 (74)	125 (74)	316 (87)	178 (85)	427 (85)	109 (84)	260 (84)	234 (87)	316 (87)	140 (84)
	Lobular	227 (18)	53 (12)	280 (17)	38 (14)	208 (16)	80 (16)	225 (16)	27 (16)	16 (4)	10 (5)	29 (6)	3 (2)	21 (7)	7 (3)	19 (5)	8 (5)
	Other	127 (10)	36 (8)	160 (10)	33 (13)	141 (11)	41 (8)	131 (9)	17 (10)	30 (8)	21 (10)	45 (9)	18 (14)	28 (9)	28 (10)	30 (8)	18 (11)
p-value		0.008		0.254		0.169		0.966		0.753		0.085*		0.060		0.616	
Grade	1	251 (23)	110 (31)	362 (26)	43 (21)	283 (26)	83 (20)	283 (24)	31 (25)	20 (6)	4 (2)	30 (7)	2 (2)	22 (8)	8 (4)	22 (7)	2 (2)
	2	624 (56)	172 (49)	781 (55)	105 (51)	594 (55)	221 (54)	655 (55)	65 (53)	92 (29)	39 (22)	131 (30)	21 (21)	78 (29)	52 (24)	90 (28)	34 (26)
	3	232 (21)	68 (19)	271 (19)	56 (27)	203 (19)	109 (26)	243 (21)	26 (21)	204 (65)	131 (75)	270 (63)	78 (77)	171 (63)	157 (72)	205 (65)	97 (73)
p-value		0.004		0.019		0.002		0.896		0.023*		0.012*		0.040		0.033*	
Node status	Negative	714 (60)	253 (64)	936 (61)	151 (63)	722 (62)	291 (61)	792 (62)	101 (65)	175 (51)	112 (60)	262 (57)	67 (54)	163 (58)	139 (54)	171 (50)	90 (60)
	Positive	479 (40)	140 (36)	599 (39)	90 (37)	441 (38)	185 (39)	494 (38)	54 (35)	169 (49)	74 (40)	201 (43)	57 (46)	119 (42)	117 (46)	168 (50)	60 (40)
p-value		0.111		0.619		0.720		0.386		0.039		0.611		0.413		0.051	
Tumour size	<2cm	703 (57)	267 (65)	929 (58)	143 (57)	727 (60)	274 (57)	776 (58)	96 (58)	153 (44)	84 (43)	206 (43)	53 (43)	130 (45)	101 (40)	145 (41)	60 (41)
	2-4.9cm	502 (41)	131 (32)	614 (39)	103 (41)	462 (38)	194 (40)	518 (39)	60 (36)	179 (52)	103 (53)	249 (52)	63 (52)	151 (52)	144 (56)	186 (53)	84 (57)
	≥5cm	31 (3)	15 (4)	46 (3)	5 (2)	32 (3)	14 (3)	36 (3)	9 (5)	13 (4)	7 (4)	20 (4)	6 (5)	9 (3)	10 (4)	19 (5)	4 (3)
p-value		0.004		0.595		0.591		0.140		0.964		0.940		0.445		0.395	

*Fisher's exact test
