# Supplementary material for: Cancer stem cell markers in breast cancer: pathological, clinical and prognostic significance
Source: Breast Cancer Res. 2011 Nov 23;13(6):R118. doi: 10.1186/bcr3061 (PMC3326560; doi:10.1186/bcr3061)
Supplement: Additional file 9 — CSC marker associations with molecular characteristics using zero as a cut-point for dichotomisation. [file bcr3061-S9.PDF]

Supplementary Table 9: CSC marker associations with molecular characteristics using zero as a cut-point for dichotomisation

		ER POSITIVE								ER NEGATIVE							
Variable		$CD44^{+}CD24^{-/low}$		ALDH1A1		ALDH1A3		ITGA6		$CD44^{+}CD24^{-/low}$		ALDH1A1		ALDH1A3		ITGA6	
		Negative	Positive	Negative	Positive	Negative	Positive	Negative	Positive	Negative	Positive	Negative	Positive	Negative	Positive	Negative	Positive
Molecular subtype	Luminal 1a	936 (82)	319 (80)	1178 (82)	181 (75)	922 (83)	350 (75)	979 (81)	107 (74)	53 (17)	16 (9)	68 (16)	9 (8)	53 (21)	20 (9)	56 (18)	14 (11)
	Other	206 (18)	82 (20)	259 (18)	59 (25)	193 (17)	118 (25)	228 (19)	38 (26)	250 (83)	162 (91)	345 (84)	106 (92)	200 (79)	213 (91)	247 (82)	116 (89)
p-value		0.287		0.016		<0.001		0.036		0.010		0.020		<0.001		0.046	
	Luminal 1b	87 (8)	58 (14)	132 (9)	30 (13)	109 (10)	54 (12)	113 (9)	19 (13)	10 (3)	7 (4)	14 (3)	5 (4)	7 (3)	10 (4)	8 (3)	5 (4)
	Other	1055 (92)	343 (86)	1305 (91)	210 (87)	1006 (90)	414 (88)	1094 (91)	126 (87)	293 (97)	171 (96)	399 (97)	110 (96)	246 (97)	223 (96)	295 (97)	125 (96)
p-value		<0.001		0.108		0.292		0.152		0.717		0.626		0.361		0.500	
	Luminal 2	119 (10)	24 (6)	127 (9)	29 (12)	84 (8)	64 (14)	115 (10)	19 (13)	10 (3)	6 (3)	14 (3)	4 (3)	9 (4)	7 (3)	14 (5)	3 (2)
	Other	1023 (90)	377 (94)	1310 (91)	211 (88)	1031 (92)	404 (86)	1092 (90)	126 (87)	293 (97)	172 (97)	399 (97)	111 (97)	244 (96)	226 (97)	289 (95)	127 (98)
p-value		0.008		0.109		<0.001		0.173		0.967		0.963		0.733		0.418*	
	HER2	NA		NA		NA		NA		75 (25)	20 (11)	69 (17)	34 (30)	49 (19)	48 (21)	70 (23)	18 (14)
	Other									228 (75)	158 (89)	344 (83)	81 (70)	204 (81)	185 (79)	233 (77)	112 (86)
p-value										<0.001		0.002		0.734		0.028	
	CBP	NA		NA		NA		NA		99 (33)	87 (49)	152 (37)	46 (40)	83 (33)	104 (45)	91 (30)	66 (51)
	Other									204 (67)	91 (51)	261 (63)	69 (60)	170 (67)	129 (55)	212 (70)	64 (49)
p-value										<0.001		0.531		0.007		<0.001	
	5NP	NA		NA		NA		NA		56 (18)	42 (24)	96 (23)	17 (15)	52 (21)	44 (19)	64 (21)	24 (18)
	Other									247 (82)	136 (76)	317 (77)	98 (85)	201 (79)	189 (81)	239 (79)	106 (82)
p-value										0.179		0.050		0.644		0.528	
Ki67	Negative	935 (77)	322 (77)	1222 (79)	175 (70)	954 (80)	330 (70)	1016 (78)	109 (71)	170 (50)	78 (40)	230 (50)	45 (36)	155 (53)	100 (40)	183 (54)	47 (32)
	Positive	272 (23)	98 (23)	326 (21)	74 (30)	243 (20)	143 (30)	279 (22)	45 (29)	171 (50)	116 (60)	232 (50)	80 (64)	138 (47)	153 (60)	154 (46)	102 (68)
p-value		0.737		0.002		<0.001		0.031		0.031		0.006		0.002		<0.001	

*Fisher's exact test
