# Supplementary material for: Cancer stem cell markers in breast cancer: pathological, clinical and prognostic significance
Source: Breast Cancer Res. 2011 Nov 23;13(6):R118. doi: 10.1186/bcr3061 (PMC3326560; doi:10.1186/bcr3061)
Supplement: Additional file 10 — Univariate survival analyses of CSC markers using zero as a cut-point for dichotomisation. [file bcr3061-S10.PDF]

Supplementary Table 10: Univariate survival analyses of CSC markers using zero as a cut-point for dichotomisation

Complete Case Analysis										
Variable	ER Positive					ER Negative				
	n	HR (95% CI)	P	T (95% CI)	P	n	HR (95% CI)	P	T (95% CI)	P
$CD44^{+}CD24^{-/low}$	1681	0.76 (0.52 - 1.1)	0.159	NA		561	1.0 (0.68 - 1.5)	0.993	NA	
ALDH1A1	1873	1.2 (0.83 - 1.8)	0.294	NA		621	1.4 (0.97 - 2.2)	0.072	NA	
ALDH1A3	1731	0.94 (0.67 - 1.3)	0.698	NA		566	1.3 (0.92 - 1.9)	0.131	NA	
ITGA6	1523	0.58 (0.30 - 1.1)	0.116	NA		519	1.3 (0.88 - 1.9)	0.190	NA	
Total CSCs	1123	0.90 (0.71 - 1.1)	0.349	NA		375	1.8 (1.2 - 2.9)	0.009	0.67 (0.47 - 0.97)	0.036
**Multiple Imputation (M=50)**										
Variable	ER Positive					ER Negative				
	n	HR (95% CI)	P	T (95% CI)	P	n	HR (95% CI)	P	T (95% CI)	P
$CD44^{+}CD24^{-/low}$	2903	0.82 (0.57 - 1.2)	0.265	NA		1070	0.99 (0.69 - 1.4)	0.974	NA	
ALDH1A1		1.2 (0.81 - 1.7)	0.422	NA			1.2 (0.86 - 1.8)	0.257	NA	
ALDH1A3		1.1 (0.80 - 1.5)	0.593	NA			1.3 (0.98 - 1.8)	0.068	NA	
ITGA6		0.79 (0.47 - 1.4)	0.398	NA			1.2 (0.83 - 1.7)	0.336	NA	
Total CSCs		0.97 (0.82 - 1.2)	0.767	NA			1.7 (1.2 - 2.4)	0.002	0.72 (0.56 - 0.94)	0.014
