# Supplementary material for: Cancer stem cell markers in breast cancer: pathological, clinical and prognostic significance
Source: Breast Cancer Res. 2011 Nov 23;13(6):R118. doi: 10.1186/bcr3061 (PMC3326560; doi:10.1186/bcr3061)
Supplement: Additional file 11 — Multivariate survival analyses for 'Total CSCs' using zero as a cut-point for dichotomisation of constituent CSC markers. [file bcr3061-S11.PDF]

## Supplementary Table 11: Multivariate survival analyses for 'Total CSCs' using zero as a cut-point for dichotomisation of constituent CSC markers

| Variable | Complete Case Analysis | | | | | Multiple Imputation (M=50) | | | | |
|---|---|---|---|---|---|---|---|---|---|---|
| | n | HR (95% CI) | P | T (95% CI) | P | n | HR (95% CI) | P | T (95% CI) | P |
| Grade | | 4.1 (1.2 - 14.1) | 0.027 | NA | | | 4.0 (1.8 - 8.6) | <0.001 | 0.41 (0.25 - 0.67) | <0.001 |
| Node status | | 4.0 (2.2 - 7.0) | <0.001 | NA | | | 2.9 (2.1 - 4.0) | <0.001 | NA | |
| Tumour size | | * | | NA | | | 1.5 (1.1 - 1.9) | 0.003 | NA | |
| PR + | 298 | 0.17 (0.06 - 0.48) | 0.001 | NA | | 1070 | 0.43 (0.26 - 0.70) | 0.001 | NA | |
| HER2 + | | * | | NA | | | 1.4 (1.0 - 2.0) | 0.035 | NA | |
| Endocrine therapy | | 1.6 (0.94 - 2.8) | 0.082 | NA | | | * | | NA | |
| **Total CSCs (0-3)** | | **1.4 (1.1 - 1.8)** | **0.017** | **NA** | | | **1.5 (1.1 - 2.1)** | **0.024** | **0.77 (0.59 - 1.0)** | **0.062** |

*Dropped from the final model
